# Circular RNA hsa_circ_0000915 promotes propranolol resistance of hemangioma stem cells in infantile haemangiomas

**DOI:** 10.1186/s40246-022-00416-w

**Published:** 2022-09-27

**Authors:** Hongrang Chen, Yongsheng Li

**Affiliations:** Department of Vascular and Thyroid Surgery, Department of General Surgery, First Affiliated Hospital of Anhui Medical University, Hefei, 230022 Anhui China

**Keywords:** Infantile haemangiomas, Propranolol resistance, Circ_0000915, miR-890, RNF187

## Abstract

**Background:**

Propranolol is a first-line clinical drug for infantile haemangiomas (IH) therapy. Nevertheless, resistance to propranolol is observed in some patients with IH. Circular RNAs (circRNAs) has been increasingly reported to act as a pivotal regulator in tumor progression. However, the underlying mechanism of circRNAs in IH remains unclear.

**Methods:**

Quantitative real-time polymerase chain reaction was performed to detect Circ_0000915, miR-890 and RNF187 expression. Protein levels were determined using western blot. CCK-8 assay was used to measure cell proliferation. Caspase-3 activity assay and flow cytometry were conducted to determine cell apoptosis. Luciferase reporter assay was carried out to assess the interaction between miR-890 and Circ_0000915 or RNF187. Chromatin immunoprecipitation assay was performed to detect the interaction between STAT3 and Circ_0000915 promoter. Biotin pull-down assay was used to detect the direct interaction between miR-890 and Circ_0000915. In vivo experiments were performed to measure tumor formation.

**Results:**

Here, we discovered depletion of Circ_0000915 increased propranolol sensitivity of haemangioma derived stem cells (HemSCs) both in vitro and in vivo, whereas forced expression of Circ_0000915 exhibited opposite effects. Mechanistically, Circ_0000915, transcriptionally induced by IL-6/STAT3 pathway, competed with RNF187 for the biding site in miR-890, led to upregulation of RNF187 by acting as a miR-890 “sponge”. Furthermore, silence of miR-890 reversed increased propranolol sensitivity of HemSCs due to Circ_0000915 ablation. Moreover, increased Circ_0000915 and RNF187 levels were observed in IH tissues and positively associated with propranolol resistance, miR-890 exhibited an inverse expression pattern.

**Conclusion:**

We thereby uncover the activation of IL-6/STAT3/Circ_0000915/miR-890/RNF187 axis in propranolol resistance of IH, and provide therapeutic implications for patients of IH with propranolol resistance.

**Supplementary Information:**

The online version contains supplementary material available at 10.1186/s40246-022-00416-w.

## Introduction

Infantile haemangioma (IH) is one of the most prevalent soft-tissue tumors, which occurs in 3% to 10% of infancy [1]. IH has been well characterized by two phases called proliferating and involuting phases: rapid growth occurs in infancy followed by slow involution in early childhood [2]. Nevertheless, some IHs exhibit a severe aggressiveness for their damage to the normal tissues and organs in the proliferating phase and even progress into a life-threatening disease [3]. Propranolol is widely used in IH’s therapy for its high safety and effectiveness in the treatment of IHs [4, 5]. However, a small percentage of patients with IHs develop resistance to propranolol treatment [6], the underlying mechanisms involved remain unclear. Plenty of studies have reported haemangioma derived stem cells (HemSCs) may be the cellular origin of IHs and play a crucial role in the progression of IHs [7–9]. We ought to explore whether HemSCs contribute to the acquisition of propranolol resistance in IHs.

Circular RNAs (circRNAs) are increasingly studied noncoding RNAs (ncRNAs) in the past years [10], which are characterized by covalently closed continuous loop with no protein-coding capacity [11]. It has been proven that circRNAs, which, by competing with other RNAs for the same miRNA binding site to form a network of posttranscriptional regulation [12], take part in multiple physiological and pathological processes recently [13–15]. Especially, circRNAs have been widely reported to serve as a pivotal mediator in carcinogenesis and tumor progression [16, 17]. For example, Chen et al. demonstrated that circTADA2As suppressed breast cancer progression and metastasis via targeting miR-203a-3p/SOCS3 axis [18]. Huang et al. reported Circular RNA circ-RanGAP1 promoted VEGFA expression by targeting miR-877-3p to facilitate gastric cancer invasion and metastasis [19]. Xu et al. showed Circular RNA hsa_circ_0000515 acted as a miR-326 sponge to promote cervical cancer progression through up-regulation of ELK1 [20]. Studies about circRNAs in IHs are emerging; the expression patterns and functional mechanisms of circRNAs in IHs are being increasingly explored [21–23].

IL-6/STAT3 signaling pathway has been widely reported to play a pivotal role in tumorigenesis and aggressiveness of multiple cancers [24], which could be regulated by important tumor-related genes to mediate tumor growth or metastasis [25, 26], could also induced expression of key cancer-related factors to promote tumor progression [27, 28], but the function of IL-6/STAT3 signaling pathway in IHs has been less explored.

In the present study, we focused on a novel circRNA: hsa_circ_0000915, derived from back-splicing of FKBP8 mRNA with a length of 259 nucleotides. We would detect the functions of Circ_0000915 in propranolol resistance of IHs both in vitro and in vivo. A specific study on mechanisms would be conducted to reveal the upstream regulators and downstream targets of Circ_0000915, which facilitated the enhanced effects of Circ_0000915 on propranolol resistance of IHs. Moreover, we would examine the expression patterns of Circ_0000915 and the upstream/downstream regulators in IHs. Our study may contribute to develop new diagnostic and therapeutic strategies for IHs patients suffering propranolol resistance.

## Materials and methods

### Cell isolation and culture

HemSCs were isolated from proliferating IH specimens. The detailed protocols of isolation and culture for HemSCs were described previously [7, 29]. Human embryonic kidney (HEK) 293 T cells were purchased from American Type Culture Collection (ATCC, Manassas, USA) and cultured in Dulbecco's modified Eagle's medium (DMEM) (Hyclone, Logan, UT) with 10% fetal bovine serum (FBS) (Hyclone), 100 units/ml penicillin, 100 units/ml streptomycin (Invitrogen, Carlsbad, CA, USA). Cells were all maintained in a humidified incubator with 5% CO_2_ at 37℃.

### Plasmid construction and cell transfection

Sequence of Circ_0000915 was amplified and inserted into PLCDH-ciR as described previously [30]. Specific siRNAs against Circ_0000915, RNF187 and STAT3, miR-890 mimics, miR-890 inhibitor and their corresponding negative control oligonucleotides were synthesized by GenePharma (Shanghai, China). Cell transfection was performed by using Lipofectamine 2000 (Invitrogen) according to the manufactural protocol. Oligonucleotides used here were all listed in Additional file 1: Table S1.

### RNA extraction and quantitative real-time PCR (qRT-PCR)

Total RNAs of IH tissues, normal skin tissues and HemSCs were extracted by using TRIzol™ Reagent (Thermo Fisher Scientific Inc., Waltham, USA) following the instruction. mRNA was reverted into complementary DNA (cDNA) with HiScript II Q RT SuperMix for qPCR (+ gDNA wiper) (Vazyme, Nanjing, China) as recommended. SuperScript IV Reverse Transcriptase (Invitrogen) was used for reverse transcription of the miRNAs in accordance with the manufacturer's instructions. qRT-PCR analysis was carried out using AceQ qPCR SYBR Green Master Mix (Vazyme, Nanjing, China) with CFX96 Touch Real-Time PCR Detection System (Bio-Rad, CA, 96 USA). The relative expression or enrichment of subjects was calculated with the 2^−ΔΔCt^ method, normalized to U6 or GAPDH. All primers used here were listed in Additional file 1: Table S1.

### Western blot analysis

Total proteins of HemSCs were extracted using RIPA lysis buffer (Solarbio Science & Technology, Beijing, China). Western blot was performed as previously described [31]. Primary antibodies including anti-Cyclin D1 (ab16663), anti-PCNA (ab29), anti-β-actin (ab8226) were purchased from Abcam, anti-STAT3 (#4904) and anti-pSTAT3 (#9131) were purchased from Cell Signaling Technology, MA, USA, and anti-RNF187 (NBP2-83456) was obtained from Novus Biologicals.

### Cell viability assay

Cell viability of HemSCs was measured by cell counting kit-8 kit (CCK-8, Sigma-Aldrich) as recommended [32]. In brief, HemSCs (10^4^ per well) with different transfection were seeded into 24-well plate and subsequently treated with various concentrations of propranolol for 72 h. 10 μl CCK-8 reagent was added into each well with 2 h incubation. A BIO-TEK ELx800 Universal Microplate Reader (Bio-Tek, Winooski, VT, USA) was used for detection of the absorbance at 450 nm/630 nm. IC_50_ value for propranolol was calculated according to cell growth curves.

### Apoptosis assay

The caspase-3 activity was detected to assess cell apoptosis of HemSCs by using Caspase-3 Colorimetric Activity Assay Kit (Beyotime, Shanghai, China) referring to the manufacturer’s guide.

To quantify cell apoptosis of HemSCs with different treatment, cells were fixed with 70% ethanol and then double stained with propidium iodide and annexin V-fluorescein isothiocyanate (FITC) according to the manufacturer’s instructions (Annexin V-FITC apoptosis detection kit I; BD, San Jose, CA, USA).

### Murine hemangioma model

The female athymic nu/nu mice (8 weeks old) were purchased from the Shanghai Experimental Animal Center of the Chinese Academy of Sciences (Shanghai, People’s Republic of China). All procedures were approved by the Committee on Animals of the First Affiliated Hospital of Anhui Medical University.

To study if Circ_0000915 mediates the effects of propranolol on HemSCs in vivo, a xenograft mouse model of infantile hemangioma was used. 1.5 × 10^6^ HemSCs suspended in Matrigel (BD Matrigel™ Basement Membrane) was implanted subcutaneously into the flanks of female nude mice (*n* = 5, day 0). Mice were treated with propranolol (10 mg/kg) by intraperitoneal injection every five days [33]. The hemangioma volume was measured and calculated using the formula: (width^2^ × length)/2 at the indicated day. On day 35, the mice were euthanized, and the hemangioma was resected and weighed.

### Subcellular fractionation

Nuclear and cytosol RNA from HemSCs was prepared and collected by using PARIS Kit (Life Technologies, Carlsbad, CA, USA) as recommended. qRT-PCR was employed to measure the relative distribution of Circ_0000915 in HemSCs. GAPDH and U6 were used as cytoplasmic and nuclear control, respectively.

### Luciferase activity assay

Luciferase activity assay was conducted to examine the interaction between different molecules. To detect the interaction between Circ_0000915 and STAT3, cells were transfected with the pGL3-based constructs containing the Circ_0000915 promoter together with Renilla luciferase plasmids. To evaluate the interaction between Circ_0000915 or RNF187 and miR-890, the wide-type and mutant Circ_0000915 (Circ_0000915-wt, Circ_0000915-mt) and RNF187 3’UTR (RNF187 3’UTR-wt, RNF187 3’UTR-mt) were cloned and inserted into the psiCHECK2 luciferase vector. Cells were co-transfected with luciferase plasmids and miR-890 mimics. Luciferase activities were examined with a dual-Glo Luciferase Assay System (Promega, USA) referring to the manual after 48 h transfection. Oligonucleotides used here were all listed in Additional file 1: Table S1.

### Chromatin immunoprecipitation (ChIP) assay

Direct binding of STAT3 to Circ_0000915 promoter was analyzed by ChIP using an EZ-ChIP Kit (Millipore) in accordance with the provider’s protocol. Sonicated chromatin was immunoprecipitated using an anti-STAT3 antibody, after which enriched fragments were detected by real-time PCR as described above. Primers of Circ_0000915 promoter were listed in Additional file 1: Table S1.

### Biotin pull-down assay

To enrich RNAs directly interacted with Circ_0000915, three specific antisense biotin-labeled probes against Circ_0000915 were incubated with HemSCs cell lysates. Their corresponding sense biotin-labeled probes were used as the negative control. Streptavidin magnetic beads (Invitrogen) were used to absorb biotin-labeled probes-enriched fractions 4 h after incubation. Beads were washed for RNA extraction and the extracted RNA was subsequently subjected to qRT-PCR analysis. Biotin-labeled oligonucleotides used here were all listed in Additional file 1: Table S1.

For biotin miRNA pull-down assay, the detailed protocol was described previously [34]. Biotinylated RNAs of miR-890 (Biotin-miR-890) and negative control (Biotin-miR-NC) were purchased from GenePharma (Shanghai, China).

### Clinical specimens

Normal skin tissues and IH tissues in proliferative phase (*n* = 50) were collected from Department of Vascular and Thyroid Surgery, the First Affiliated Hospital of Anhui Medical University from 2015 to 2019. Patient information is listed in Additional file 2: Table S2. Samples were maintained in liquid nitrogen immediately after resection. Informed consents were signed by each patient enrolled. The experimental protocols were approved by the Ethics Committees of the First Affiliated Hospital of Anhui Medical University.

### Statistical analysis

All data from three independent experiments at least was shown as mean ± standard deviation (SD) unless otherwise stated. GraphPad Prism 8.0 was employed for data processing and analysis. Student’s *t*-test or two-way ANOVA was used to analyze the difference between two groups. *p* < 0.05 was considered as statistically significant.

## Results

### Circ_0000915 reduced propranolol sensitivity of HemSCs

**Fig. 1:**
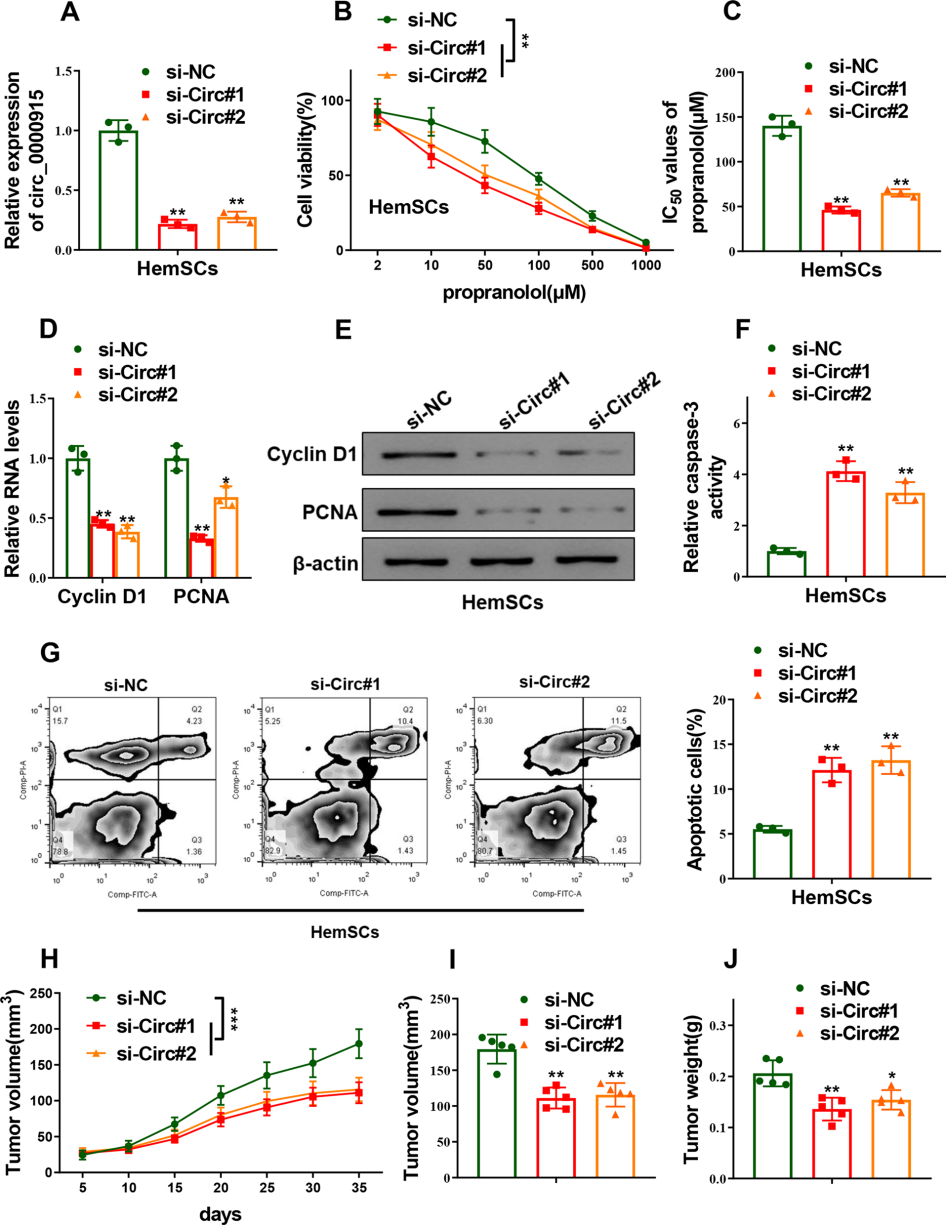
Silence of Circ_0000915 enhanced propranolol sensitivity of HemSCs. **A** Expression of Circ_0000915 was quantified by qRT-PCR in HemSCs transfected with specific siRNAs against Circ_0000915 (si-Circ#1 and si-Circ#2) or siRNA negative control (si-NC). **B** CCK-8 assay showed cell viability of HemSCs exposed to different concentration of propranolol for 72 h after transfection with si-Circ#1, si-Circ#2 or si-NC. **C** IC_50_ value of propranolol (72 h treatment) was calculated after HemSCs were transfected with si-Circ#1, si-Circ#2 or si-NC. **D, E** Expression of proliferative-related markers (Cyclin D1 and PCNA) in Circ_0000915-silent HemSCs exposed to propranolol (20 μM) for 48 h was measured by qRT-PCR and Western blot. **F, G** Cell apoptosis of Circ_0000915-silent HemSCs exposed to propranolol (20 μM) for 48 h were determined by caspase-3 activity assay (**F**) and Annexin-V/PI double staining assay (**G**). The tumor growth curve (**H**), the tumor volume (**I**) and weight (**J**) at the end point (day 35) derived from Circ_0000915-silent HemSCs. Data are presented as mean ± S.D from three independent experiments. *P < 0.05; **P < 0.01; ***P < 0.001. (Two-way ANOVA for B and H, Student’s t-test for others)

In an attempt to explore the effects of Circ_0000915 on propranolol resistance of HemSCs, two specific siRNAs against Circ_0000915 (si-Circ#1 and si-Circ#2) were transfected into HemSCs to knockdown the endogenic Circ_0000915 (*p = *0.0013 for si-Circ#1 and *p* = 0.0010 for si-Circ#2, Fig. 1A). CCK-8 assay showed silence of Circ_0000915 significantly decreased cell viability of HemSCs with the treatment of indicated dose of propranolol (*p < *0.0001 for si-Circ#1 and *p* = 0.0027 for si-Circ#2, Fig. 1B), IC_50_ values of propranolol obviously reduced in Circ_0000915-depleted HemSCs as compared with the negative control counterpart (*p = *0.0021 for si-Circ#1 and *p* = 0.0034 for si-Circ#2, Fig. 1C). Knockdown of Circ_0000915 inhibited the expression of proliferative-related markers (Cyclin D1 and PCNA) at both mRNA (Cyclin D1: *p = *0.0074 for si-Circ#1 and *p* = 0.0026 for si-Circ#2; PCNA: *p = *0.0053 for si-Circ#1 and *p* = 0.0158 for si-Circ#2, Fig. 1D) and protein levels (Fig. 1E). Moreover, cell apoptosis of HemSCs induced by propranolol exposure was enhanced with Circ_0000915 ablation, as determined by Caspase-3 activity assay (*p = *0.0027 for si-Circ#1 and *p* = 0.0067 for si-Circ#2, Fig. 1F) and Annexin-V/PI double staining assay (*p = *0.0099 for si-Circ#1 and *p* = 0.0098 for si-Circ#2, Fig. 1G). Furthermore, hemangioma derived from Circ_0000915-depleted HemSCs exhibited smaller mean volume (*p < *0.0001 for si-Circ#1 and *p < *0.0001 for si-Circ#2, Fig. 1H and p = 0.0004 for si-Circ#1 and *p* = 0.0007 for si-Circ#2, Fig. 1I) and lower weight (*p* = 0.0018 for si-Circ#1 and *p* = 0.0074 for si-Circ#2, Fig. 1J).

**Fig. 2:**
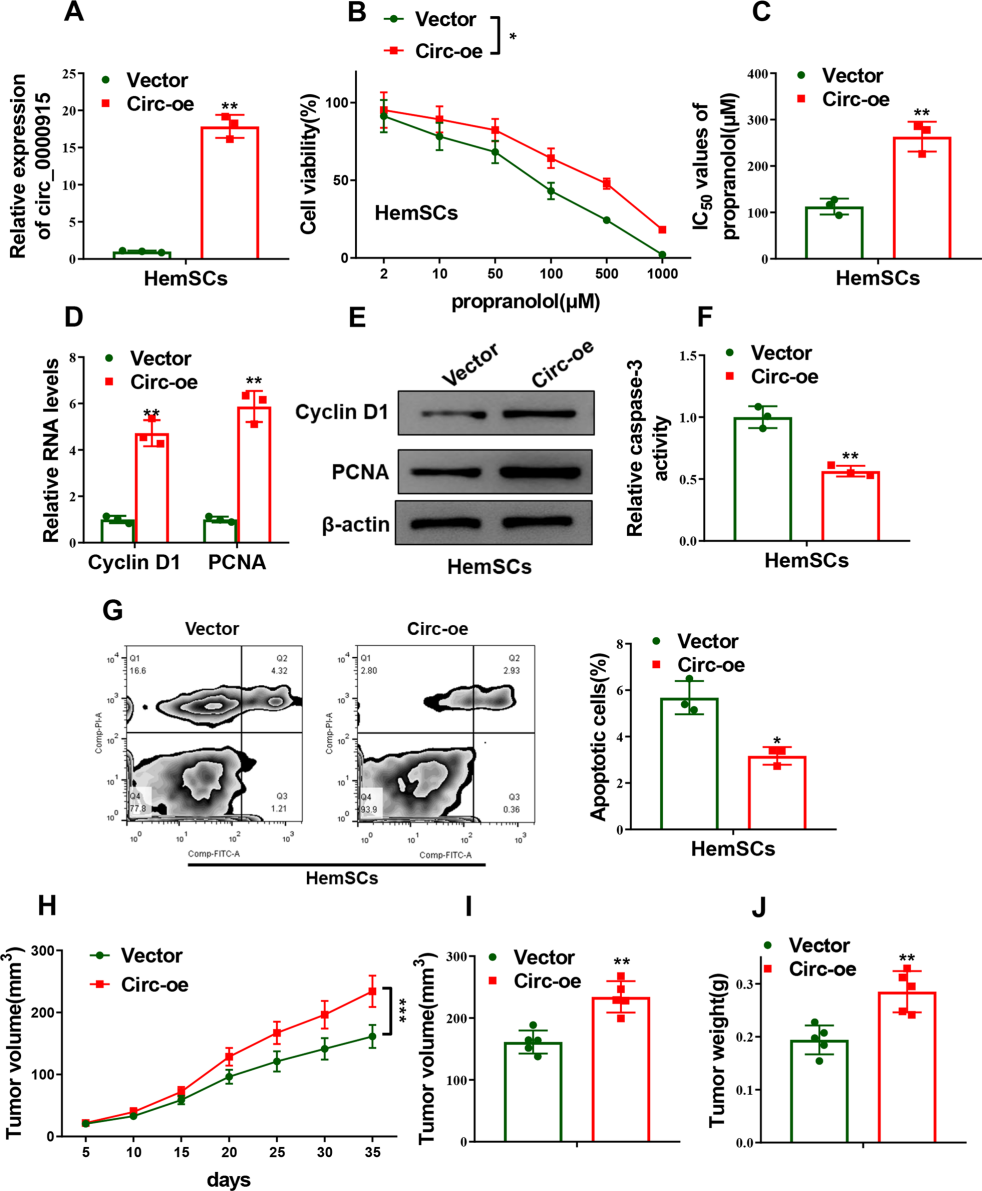
Ectopic expression of Circ_0000915 promoted propranolol resistance of HemSCs. **A** qRT-PCR confirmed the overexpression efficiency of Circ_0000915 in HemSCs transfected with Circ_0000915 plasmid (Circ-oe) or empty vector (Vector). **B** CCK-8 assay showed cell viability of HemSCs exposed to different concentration of propranolol for 72 h after transfection with Circ-oe or Vector. **C** IC_50_ value of propranolol (72 h treatment) was calculated after HemSCs were transfected with Circ-oe or Vector. **D**, **E** Expression of proliferative-related markers (Cyclin D1 and PCNA) in Circ_0000915-overexpressing HemSCs exposed to propranolol (20 μM) for 48 h was measured by qRT-PCR and Western blot. **F**, **G** Cell apoptosis of Circ_0000915-overexpressing HemSCs exposed to propranolol (20 μM) for 48 h were determined by caspase-3 activity assay (**F**) and Annexin-V/PI double staining assay (**G**). The tumor growth curve (**H**), the tumor volume (**I**) and weight (**J**) at the end point (day 35) derived from Circ_0000915-overexpressing HemSCs. Data are presented as mean ± S.D from three independent experiments. *P < 0.05; **P < 0.01; ***P < 0.001. (Two-way ANOVA for B and H, Student’s t-test for others)

On the other hand, gain-of-function experiments were subsequently conducted to verify the effects of Circ_0000915 overexpression on propranolol sensitivity of HemSCs. We first confirmed the transfection efficiency of Circ_0000915 plasmid (Circ-oe) in HemSCs by qRT-PCR (*p = *0.0027, Fig. 2A). Overexpression of Circ_0000915 promoted cell viability of HemSCs with the treatment of indicated dose of propranolol (*p = *0.0148, Fig. 2B), increased IC_50_ values of propranolol (*p* = 0.0054, Fig. 2C) and enhanced the expression of Cyclin D1 and PCNA (Cyclin D1: *p = *0.0048; PCNA: *p = *0.0050, Fig. 2D, E). Forcing expression of Circ_0000915 decreased cell apoptosis of HemSCs treated with propranolol (*p = *0.0050, Fig. 2F and *p* = 0.0123, Fig. 2G). In murine hemangioma model, bigger mean tumor volume (*p* < 0.0001, Fig. 2H and *p* = 0.0011, Fig. 2I) and higher tumor weight (*p* = 0.0034, Fig. 2J) were observed in hemangioma derived from Circ_0000915-overexpressing HemSCs. Taken together, these results suggested Circ_0000915 decreased propranolol sensitivity of HemSCs both in vitro and in vivo.

### Circ_0000915 was transcriptionally regulated by IL-6/STAT3 pathway

**Fig. 3:**
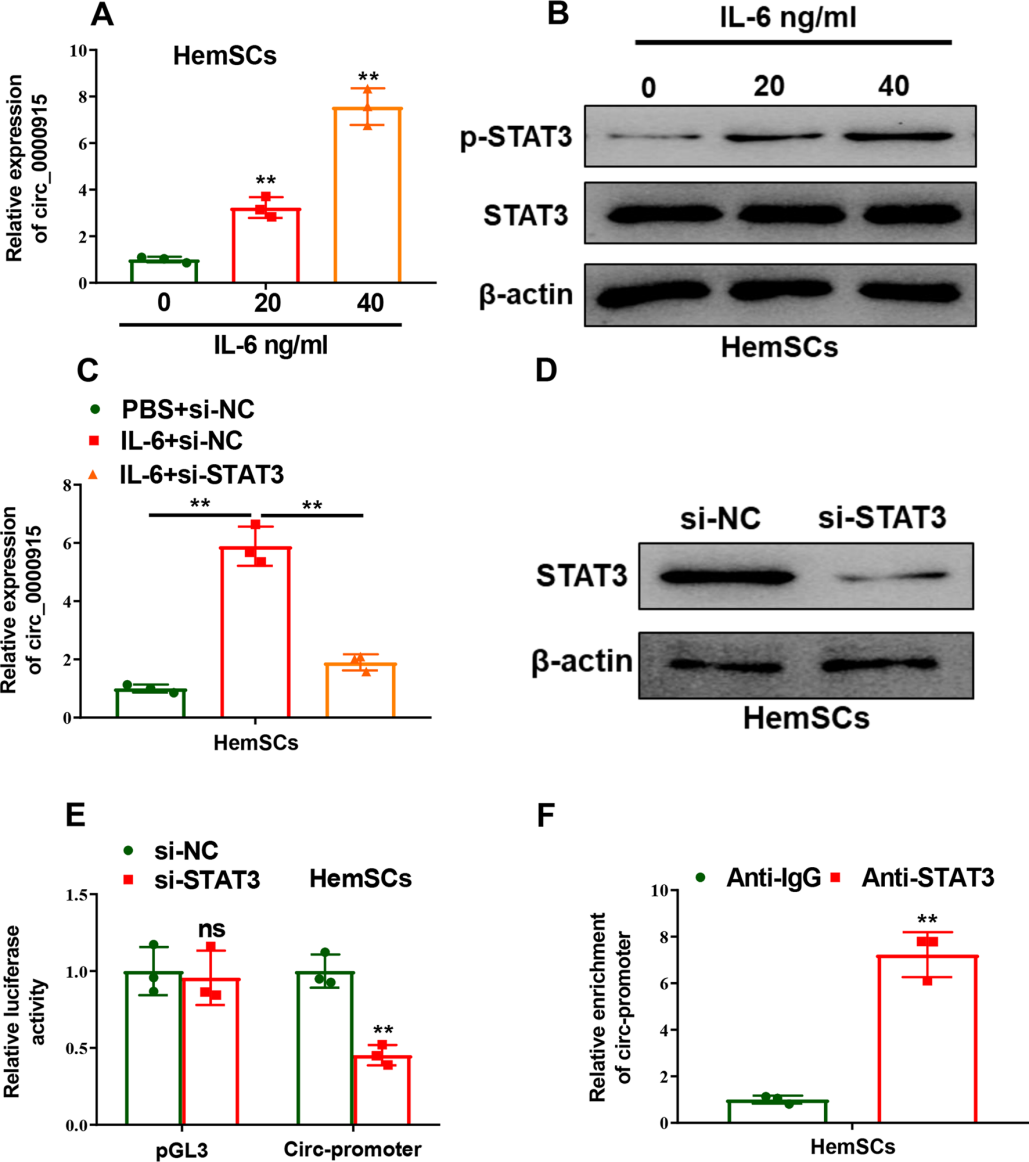
IL-6 induced Circ_0000915 expression via activation of STAT3. **A** Expression of Circ_0000915 in HemSCs treated with the indicated concentration of IL-6 for 24 h was detected by qRT-PCR. **B** STAT3 phosphorylation in HemSCs treated with the indicated concentration of IL-6 for 24 h was examined by Western blot. **C** qRT-PCR analysis of Circ_0000915 expression in HemSCs cells transfected with STAT3 siRNAs (pool) or negative control siRNAs, followed by treatment with 40 ng/ml IL-6 or PBS for 24 h. **D** Knockdown efficiency of STAT3 in HemSCs cells transfected with STAT3 siRNAs (pool) or negative control siRNAs was confirmed by Western blot. **E** HemSCs was co-transfected with either STAT3 siRNAs (pool) or negative control siRNAs plus the Circ_0000915 promoter reporter constructs and Renilla luciferase plasmid. 48 h after transfection, reporter activity was measured and plotted after normalizing with respect to Renilla luciferase activity. **F** ChIP assays in HemSCs using a STAT3 antibody, followed by qRT-PCR analysis of Circ_0000915 promoter enrichment. Data are presented as mean ± S.D from three independent experiments. *P < 0.05; **P < 0.01; ns = not significant. Student’s t-test

We subsequently investigated the regulatory mechanisms of Circ_0000915 involved after its functions in IHs were validated. Molecules which mediated expression of Circ_0000915 upstream were first explored. Potential transcription factors targeting promoter of FKBP8 were analyzed via rVista 2.0 (https://rvista.dcode.org/instr_rVISTA.html), STAT3 was included. We thereby further investigated if STAT3 transcriptionally regulated expression of Circ_0000915 indeed. HemSCs was treated with recombinant IL-6 for 24 h, Circ_0000915 was up-regulated in an IL-6 dose-dependent manner (*p = *0.0090 for 20 ng/ml and *p* = 0.0040 for 40 ng/ml, Fig. 3A), and so was the expression level of phosphorylated STAT3 (Fig. 3B). The markedly increased Circ_0000915 induced by IL-6 could be abrogated by transfection with STAT3-specific siRNAs (*p* = 0.0048 for IL-6 + si-NC and *p* = 0.0040 for IL-6 + si-STAT3, Fig. 3C and Fig. 3D). Besides, we found that Circ_0000915 promoter-fused luciferase activity was significantly inhibited upon STAT3 knockdown in HemSCs (*p* = 0.0034, Fig. 3E). Furthermore, we examined direct binding of STAT3 to Circ_0000915 promoter by ChIP assay, and noted significant enrichment of Circ_0000915 promoter fragments after STAT3 immunoprecipitation in HemSCs (*p = *0.0066, Fig. 3F). Collectively, these results indicated that Circ_0000915 was transcriptionally regulated by IL-6-activated STAT3.

### Circ_0000915 suppressed miR-890 expression by acting as a sponge

**Fig. 4:**
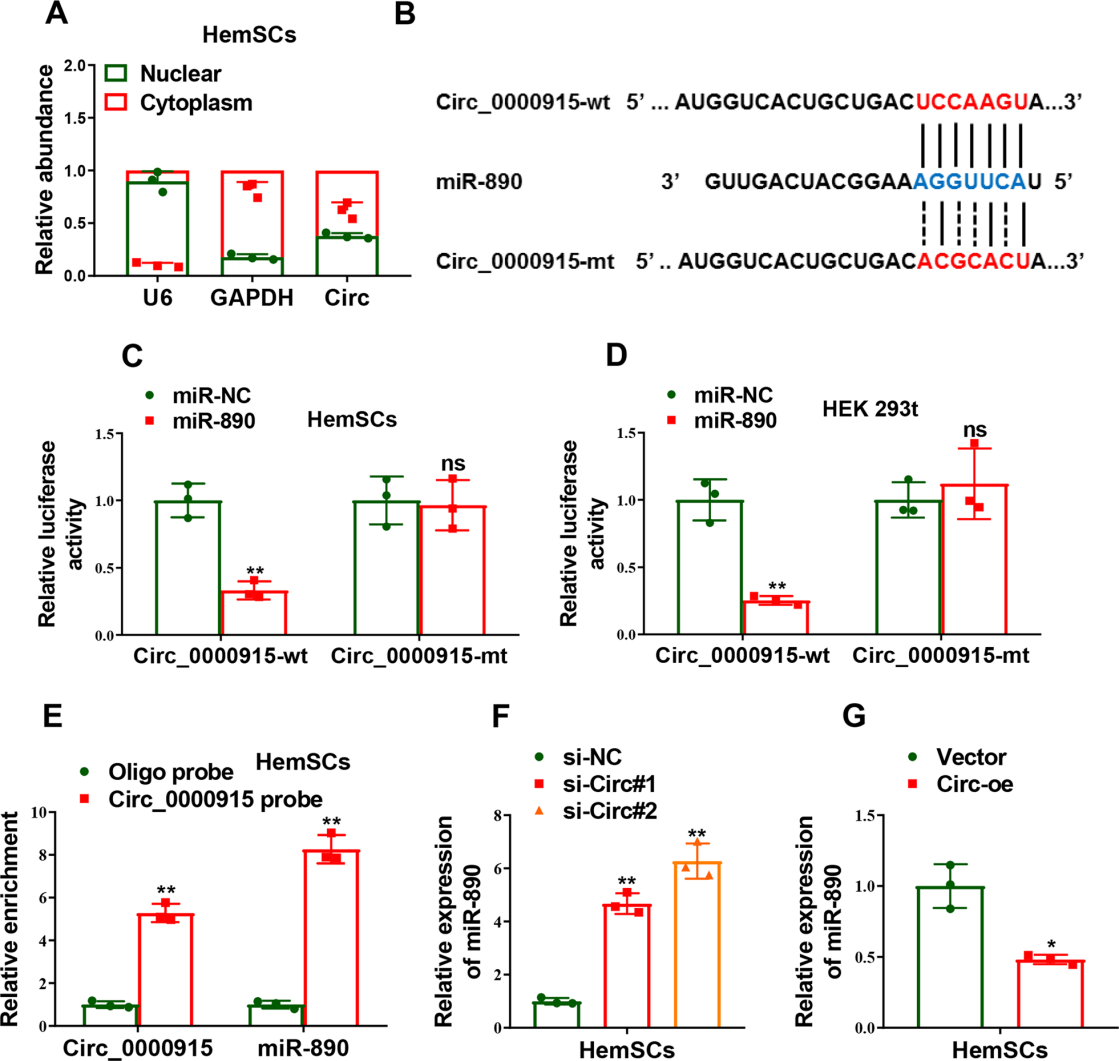
Circ_0000915 negatively regulated the expression of miR-890 in HemSCs. **A** Distribution of Circ_0000915 in nucleus and cytoplasm of HemSCs was confirmed by qRT-PCR. **B** The prediction for miR-890 binding sites on Circ_0000915 and schematic of luciferase reporter vector constructs Circ_0000915 wild-type (Circ_0000915-wt) and the miR-890-binding-site mutated (Circ_0000915-mt) one. **C**, **D** The luciferase activities in HemSCs and HEK 293t cells co-transfected with miR-890 or miR-NC mimics and luciferase reporters containing Circ_0000915-wt or Circ_0000915-mt. **E** Lysates from HemSCs were incubated with in vitro-synthesized biotin-labeled sense (Oligo probe) or antisense (Circ_0000915 probe) DNA probes against Circ_0000915 for biotin pull-down assay, followed by qRT-PCR analysis to examine Circ_0000915 and miR-890 levels. **F**,** G** Expression of miR-890 was measured by qRT-PCR in Circ_0000915-silent (**F**) and Circ_0000915-overexpressing (**G**) HemSCs. Data are presented as mean ± S.D from three independent experiments. *P < 0.05; **P < 0.011; ns = not significant. Student’s t-test

We next explored the target molecules of Circ_0000915 downstream, which largely depends on its cellular sublocalization. Firstly, we demonstrated that Circ_0000915 resided predominantly in the cytoplasm in HemSCs by qRT-PCR analysis of nuclear and cytoplasmic RNAs (Fig. 4A), which strengthened the “ceRNA” regulatory pattern [35, 36] of Circ_0000915. We then predicted the potential target miRNAs of Circ_0000915 with Circular RNA Interactome (https://circinteractome.nia.nih.gov/), and miR-890 was chosen for further study due to its functional correlation. Luciferase reporter vectors containing the wild-type (Circ_0000915-wt) and mutant (Circ_0000915-mt) miR-890 binding site within Circ_0000915 were constructed (Fig. 4B), a dramatical inhibition of luciferase activity was detected in HemSCs and HEK 293t cells co-transfected with miR-890 mimics and Circ_0000915-wt, but not with miR-890 mimics and Circ_0000915-mt (*p = *0.0036, Fig. 4C and *p* = 0.0096, Fig. 4D). To further consolidate the direct interaction between Circ_0000915 and miR-890, biotin-RNA pulldown assay was carried out and qRT-PCR analyses revealed Circ_0000915 and miR-890 were both abundantly enriched by biotin-labeled antisense probes against Circ_0000915 (Circ_0000915: *p = *0.0013; miR-890: *p = *0.0016, Fig. 4E). Besides, expression of miR-890 was significantly increased with Circ_0000915 depletion in HemSCs (*p = *0.0019 for si-Circ#1 and *p* = 0.0041 for si-Circ#2, Fig. 4F). In contrast, Ectopic Circ_0000915 expression dramatically decreased miR-890 expression level in HemSCs (*p* = 0.0242, Fig. 4G). Collectively, our data indicated Circ_0000915 decreased the expression level of endogenous miR-890 by acting as a “sponge” in HemSCs.

### RNF187 was targeted by miR-890

**Fig. 5:**
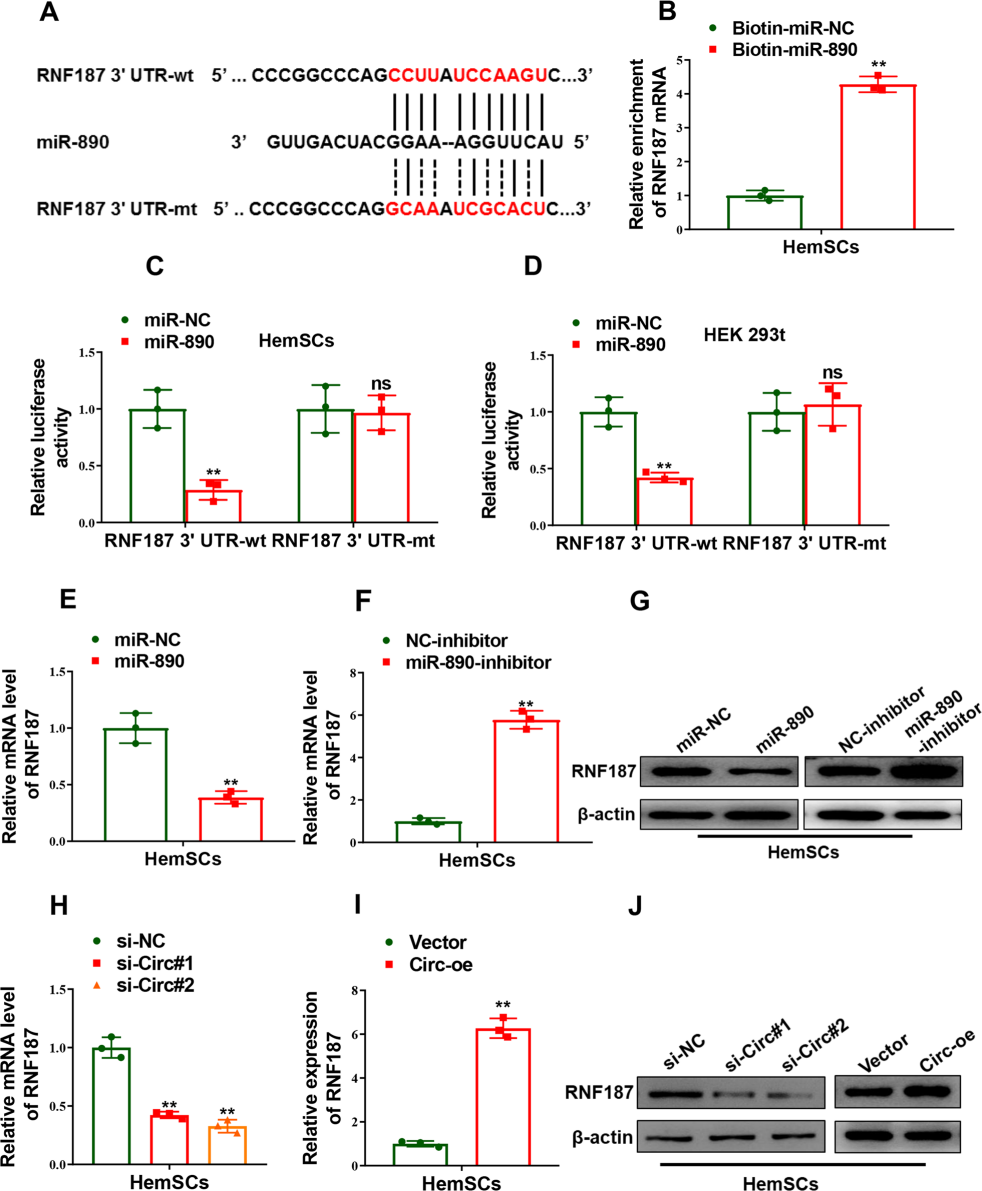
Circ_0000915 up-regulated RNF187 via inhibition of miR-890 in HemSCs. **A** The prediction for miR-890 binding sites on RNF187 transcripts and schematic of luciferase reporter vector constructs RNF187 3’UTR wild-type (RNF187 3’UTR-wt) and the miR-890-binding-site mutated (RNF187 3’UTR-mt) one. **B** Biotin-coupled miR-890 (Biotin-miR-890) captured a fold change of RNF187 mRNA in the complex as compared with biotin-coupled miR-NC (Biotin-miR-NC) in biotin-coupled miRNA capture in HemSCs. **C**, **D** The luciferase activities in HemSCs and HEK 293t cells co-transfected with miR-890 or miR-NC mimics and luciferase reporters containing RNF187 3’UTR-wt or RNF187 3’UTR-mt. **E–G** Expression of RNF187 in HemSCs transfected with miR-890 mimics and miR-890 inhibitor or their corresponding negative control was measured by qRT-PCR and Western blot. **H–J** Expression of RNF187 in Circ_0000915-silent and Circ_0000915-overexpressing HemSCs was measured by qRT-PCR and Western blot. Data are presented as mean ± S.D from three independent experiments. *P < 0.05; **P < 0.011; ns = not significant. Student’s *t*-test

Next, we seek for potential target genes of miR-890 by bioinformatics using targetscan (http://www.targetscan.org/vert_71/). RNF187 was screened as a novel function-related target of miR-890 and the alignment between miR-890 and RNF187 3’UTR was illustrated in Fig. 5A. Then we verified the interplay between miR-890 and RNF187. Firstly, we found RNF187 mRNA was significantly enriched in the complex pulled down by biotinylated miR-890 mimics (Biotin-miR-890) compared with biotinylated negative control mimics (Biotin-miR-NC), as determined by biotin miRNA pull-down assay (*p = *0.0001, Fig. 5B). Secondly, luciferase activity driven by the reporter vector containing wild-type RNF187 3’UTR (RNF187 3’UTR-wt) was notably suppressed in both HemSCs and HEK 293t cells co-transfected with miR-890 mimics, which was completely abolished when co-transfected with reporter vector containing mutant RNF187 3’UTR (RNF187 3’UTR-mt) and miR-890 mimics (*p = *0.0072, Fig. 5C and *p* = 0.0099, Fig. 5D). Furthermore, as shown in Fig. 5E–G, both mRNA and protein levels decreased in HemSCs after forced expression of miR-890 (*p = *0.0076, Fig. 5E, G) and increased after miR-890 inhibition (*p = *0.0010, Fig. 5F, G). Moreover, in accordance with the competitive regulatory effect of Circ_0000915 on miR-890, expression of RNF187 markedly down-regulated in the Circ_0000915-silenced HemSCs (*p* = 0.0043 for si-Circ#1 and *p* = 0.0009 for si-Circ#2, Fig. 5H, J), and up-regulated in HemSCs with overexpression of Circ_0000915 (*p* = 0.0012, Fig. 5I, J), at both mRNA and protein levels. In summary, these results indicated Circ_0000915 sponged miR-890 and alleviated its inhibitory effect on RNF187 expression in HemSCs.

**Fig. 6:**
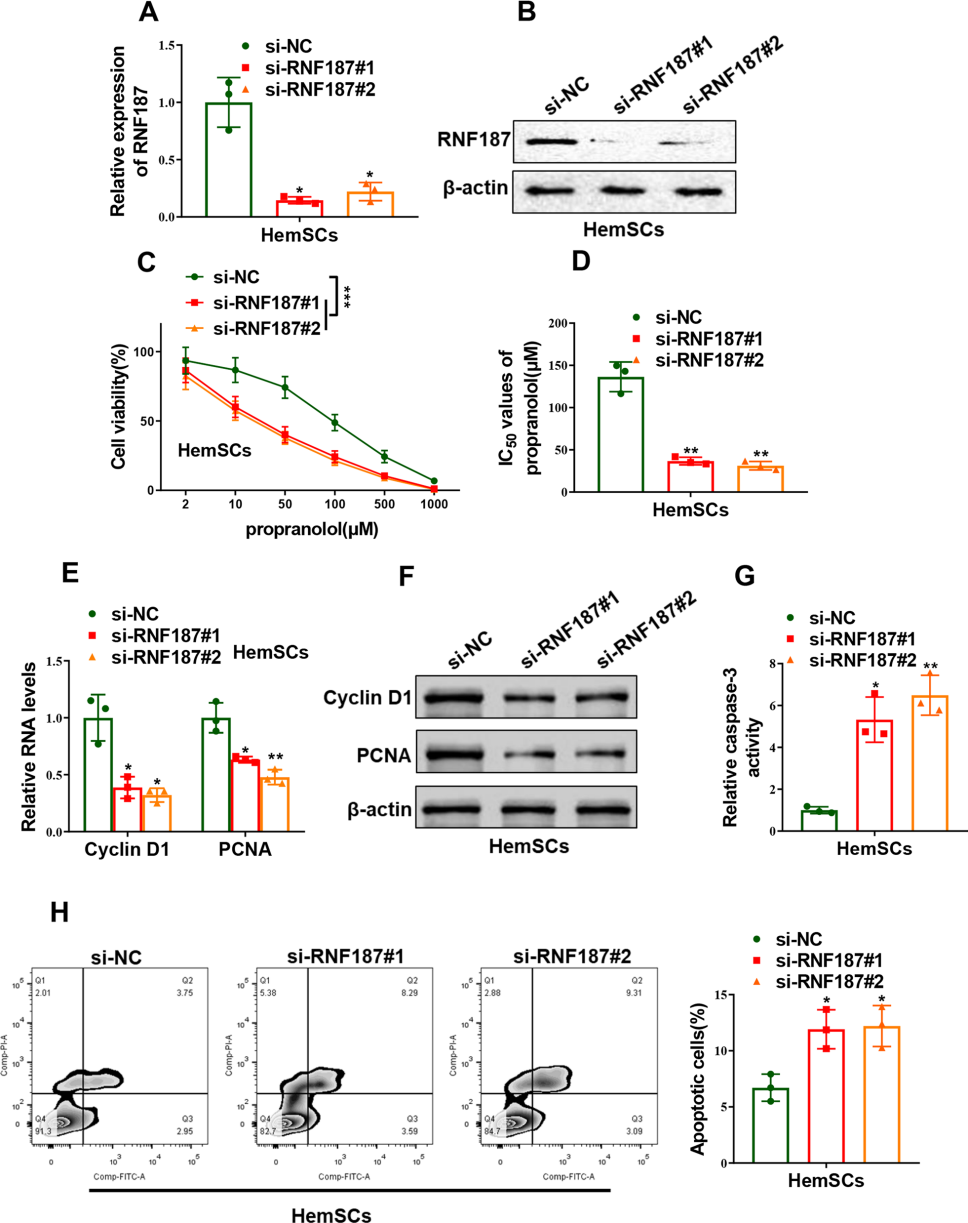
Depletion of RNF187 impaired propranolol resistance of HemSCs. **A**,** B** mRNA and protein levels of RNF187 in HemSCs transfected with specific siRNAs against RNF187 (si-RNF187#1 and si-RNF187#2) or siRNA negative control (si-NC) were detected qRT-PCR and Western blot, respectively. **C** CCK-8 assay showed cell viability of HemSCs exposed to different concentration of propranolol for 72 h after transfection with si-RNF187#1, si-RNF187#2 or si-NC. **D** IC_50_ value of propranolol (72 h treatment) was calculated after HemSCs were transfected with si-RNF187#1, si-RNF187#2 or si-NC. **E**, **F** Expression of proliferative-related markers (Cyclin D1 and PCNA) in RNF187-silent HemSCs exposed to propranolol (20 μM) for 48 h was measured by qRT-PCR and Western blot, respectively. **G**, **H** Cell apoptosis of RNF187-silent HemSCs exposed to propranolol (20 μM) for 48 h were determined by caspase-3 activity assay (**G**) and Annexin-V/PI double staining assay (**H**). *P < 0.05; **P < 0.01; ***P < 0.001. (Two-way ANOVA for C, Student’s t-test for others)

We consequently explored if RNF187 mediated propranolol sensitivity of HemSCs. Endogenic RNF187 in HemSCs was significantly silenced with transfection of specific siRNAs against RNF187 (*p = *0.0192 for si-RNF187#1 and *p* = 0.0158 for si-RNF187#2, Fig. 6A, B). In line with Circ_0000915, knockdown of RNF187 obviously impaired cell viability of HemSCs with the treatment of indicated dose of propranolol, and IC_50_ values of propranolol remarkably decreased in RNF187-depleted HemSCs as compared with the negative control counterpart, as determined by CCK-8 assay (*p < *0.0001 for si-RNF187#1 and *p < *0.0001 for si-RNF187#2, Fig. 6C and *p* = 0.0072 for si-RNF187#1 and *p* = 0.0058 for si-RNF187#2, Fig. 6D). Moreover, depletion of RNF187 observably decreased both mRNA (Cyclin D1: *p* = 0.0202 for si-RNF187#1 and *p* = 0.0212 for si-RNF187#2; PCNA: *p* = 0.0360 for si-RNF187#1 and *p* = 0.0094 for si-RNF187#2, Fig. 6E) and protein levels (Fig. 6F) of proliferative-related markers (Cyclin D1 and PCNA). Furthermore, RNF187 ablation promoted cell apoptosis rate of HemSCs induced by propranolol exposure. (*p = *0.0184 for si-RNF187#1 and *p = *0.0084 for si-RNF187#2, Fig. 6G and *p* = 0.0166 for si-RNF187#1 and *p* = 0.0167 for si-RNF187#2, Fig. 6H). Collectively, these data suggested RNF187 positively mediated propranolol resistance of HemSCs.

### Circ_0000915 mediated propranolol sensitivity of HemSCs via miR-890/RNF187 axis

**Fig. 7:**
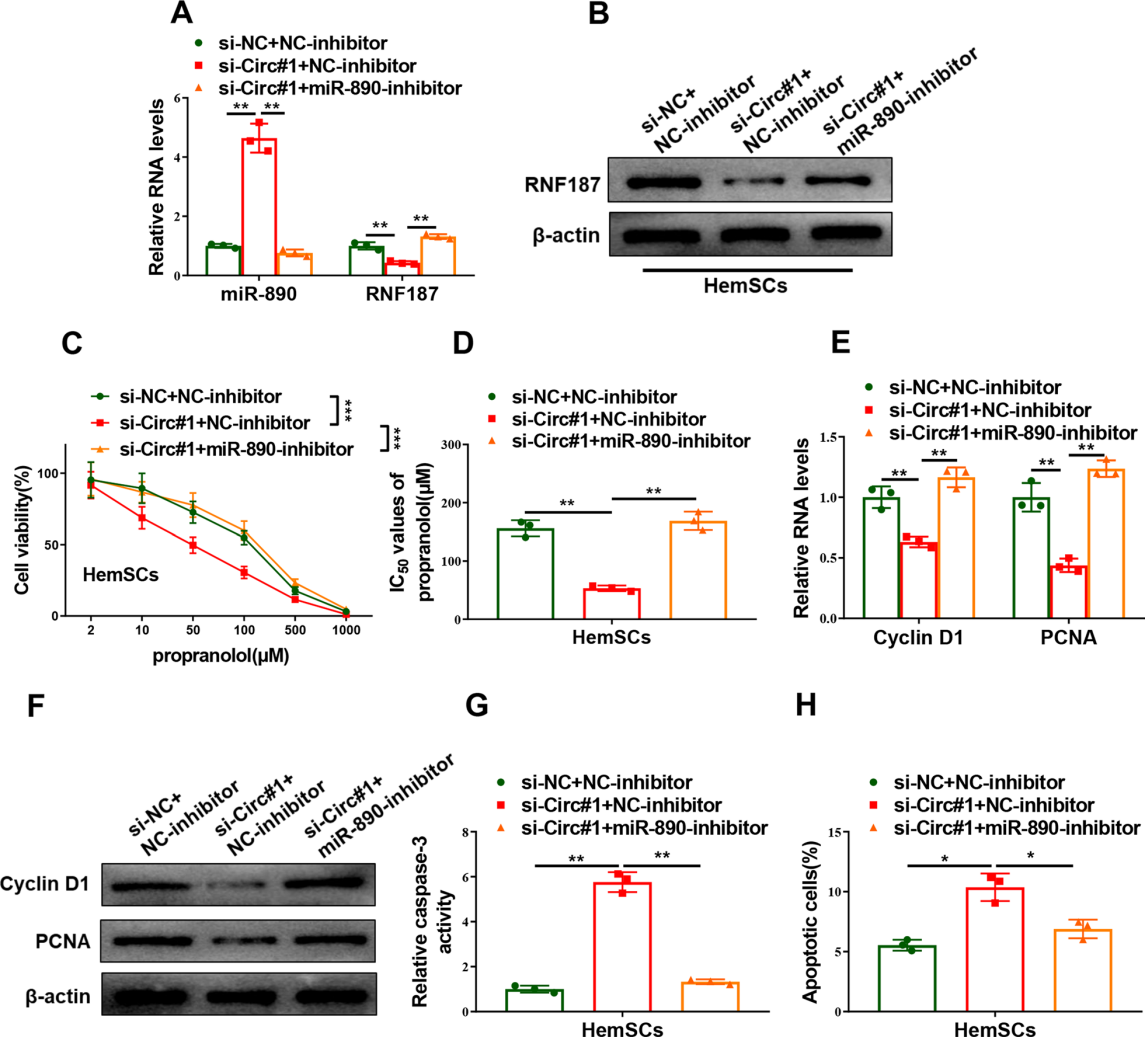
Effects of Circ_0000915 on propranolol resistance of HemSCs depended on miR-890/RNF187 pathway. HemSCs were co-transfected with si**-**NC + NC-inhibitor, si-Circ#1 + NC-inhibitor or si-Circ#1 + miR-890-inhibitor. **A** RNA levels of miR-890 and RNF187 were examined by qRT-PCR in these co-transfected HemSCs. **B** Protein level of RNF187 in these co-transfected HemSCs was detected by Western blot. **C** CCK-8 assay showed cell viability of these co-transfected HemSCs exposed to different concentration of propranolol for 72 h. **D** IC_50_ value of propranolol (72 h treatment) was calculated after HemSCs were subjected to the co-transfection. **E**, **F** Expression of proliferative-related markers (Cyclin D1 and PCNA) in these co-transfected HemSCs exposed to propranolol (20 μM) for 48 h was measured by qRT-PCR (**E**) and Western blot (**F**). **G**, **H** Cell apoptosis of these co-transfected HemSCs exposed to propranolol (20 μM) for 48 h were determined by caspase-3 activity assay (**G**) and Annexin-V/PI double staining assay (**H**). Data are presented as mean ± S.D from three independent experiments. *P < 0.05; **P < 0.01; ***P < 0.001. (Two-way ANOVA for C, Student’s t-test for others)

We have confirmed Circ_0000915 decreased propranolol sensitivity of HemSCs, but the relevance of the Circ_0000915/miR-890/RNF187 axis in propranolol resistance of HemSCs remained elusive. It was observed that the increased miR-890 and decreased RNF187 caused by Circ_0000915 depletion were obviously reverted following co-transfection with miR-890-inhibitor in HemSCs (miR-890: *p = *0.0052 for si-Circ#1 + NC-inhibitor and *p* = 0.0037 for si-Circ#1 + miR-890-inhibitor; RNF187: *p = *0.0068 for si-Circ#1 + NC-inhibitor and *p* = 0.0002 for si-Circ#1 + miR-890-inhibitor, Fig. 7A, B). Functionally, CCK-8 assay showed Circ_0000915 knockdown-induced reduced proliferation (*p < *0.0001 for si-Circ#1 + NC-inhibitor and *p < *0.0001 for si-Circ#1 + miR-890-inhibitor, Fig. 7C) and IC_50_ value of propranolol (*p* = 0.0028 for si-Circ#1 + NC-inhibitor and *p* = 0.0035 for si-Circ#1 + miR-890-inhibitor, Fig. 7D) in HemSCs were dramatically abolished by miR-890 depletion. Expression of proliferative-related markers (Cyclin D1 and PCNA) in HemSCs were markedly decreased after Circ_0000915 depletion and subsequently increased by miR-890 co-transfection (Cyclin D1: *p = *0.0084 for si-Circ#1 + NC-inhibitor and *p* = 0.0020 for si-Circ#1 + miR-890-inhibitor; PCNA: *p = *0.0060 for si-Circ#1 + NC-inhibitor and *p* = 0.0001 for si-Circ#1 + miR-890-inhibitor, Fig. 7E, F). In addition, Caspase-3 activity assay and Annexin-V/PI double staining assay revealed miR-890 ablation inhibited the apoptosis-promoting effect of Circ_0000915 knockdown in HemSCs with the treatment of propranolol (*p = *0.0011 for si-Circ#1 + NC-inhibitor and *p* = 0.0021 for si-Circ#1 + miR-890-inhibitor, Fig. 7G and p = 0.0102 for si-Circ#1 + NC-inhibitor and *p* = 0.0164 for si-Circ#1 + miR-890-inhibitor, Fig. 7H). Taken together, these results demonstrated that Circ_0000915 decreased propranolol sensitivity of HemSCs via regulating miR-890/RNF187 pathway.

### Circ_0000915, miR-890 and RNF187 were dysregulated and associated with propranolol resistance in hemangiomas

**Fig. 8:**
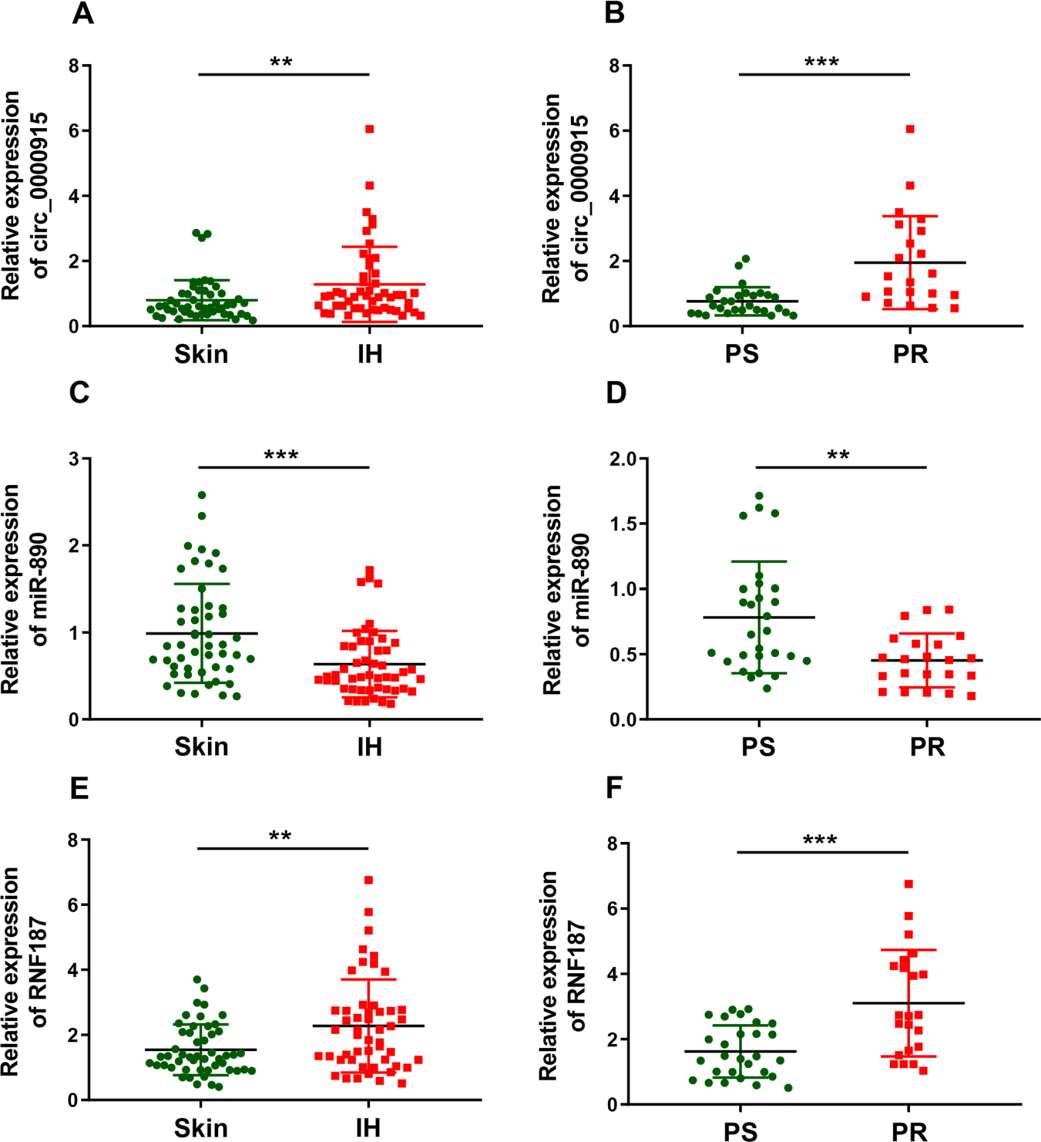
Circ_0000915, miR-890 and RNF187 were dysregulated and associated with propranolol resistance in hemangiomas. **A** Expression of Circ_0000915 was quantified by qRT-PCR in 50 paired normal skin tissues (Skin) and proliferating IH tissues (IH). **B** Expression of Circ_0000915 in IH tissues from 28 propranolol-sensitive (PS) patients and 22 propranolol-resistant (PR) patients was measured by qRT-PCR. **C** Expression of miR-890 was quantified by qRT-PCR in 50 paired normal skin tissues (Skin) and proliferating IH tissues (IH). **D** Expression of miR-890 in IH tissues from 28 propranolol-sensitive (PS) patients and 22 propranolol-resistant (PR) patients was measured by qRT-PCR. **E** Expression of RNF187 was quantified by qRT-PCR in 50 paired normal skin tissues (Skin) and proliferating IH tissues (IH). **F** Expression of RNF187 in IH tissues from 28 propranolol-sensitive (PS) patients and 22 propranolol-resistant (PR) patients was measured by qRT-PCR. *P < 0.05; **P < 0.01; ***P < 0.001. Student’s *t*-test

To strengthen the clinical significance of our study, 50 normal skin tissues and IH tissues in proliferative phase were collected. Expression levels of Circ_0000915, miR-890 and RNF187 in these samples were examined by qRT-PCR. Circ_0000915 was significantly up-regulated in IH tissues as compared with normal skin tissues (*p = *0.0095, Fig. 8A). Furthermore, expression levels of Circ_0000915 were much higher in IH tissues from propranolol-resistant patients than propranolol-sensitive ones (*p = *0.0001, Fig. 8B). Conversely, miR-890 exhibited an opposite expression pattern in IH tissues (*p = *0.0004, Fig. 8C) and was negatively associated with propranolol resistance of patients with IH (*p* = 0.0017, Fig. 8D). In line with Circ_0000915, elevated RNF187 was also observed in IH tissues (*p* = 0.0020, Fig. 8E) and positively correlated to propranolol resistance of patients with IHs (*p* = 0.0005, Fig. 8F). Collectively, these data indicated Circ_0000915 and RNF187 may play oncogenic roles and promote propranolol resistance in IHs, while miR-890 may act as a tumor suppressor and inhibit propranolol resistance in IHs.

## Discussion

IH, a type of benign vascular tumor, just affects nearly 3% to 10% of infants and young children. Nevertheless, tumors with rapid growth could still lead to serious morbidity, multiple complications, and even mortality [1, 3]. Due to a high safety and efficiency, propranolol was used as a first-line drug for IH’s therapy [37]. However, propranolol resistance has developed in few cases of IHs patients [6, 38]. Thereby, it has a profound significance to explore the underlying mechanisms contributed to propranolol resistance in IHs.

HemSCs has attracted much attention of investigators for its critical role in IHs [7]. It’s reported that CD133^+^ HemSCs differentiated into adipogenic cells during the involuting phase and led to formation of fibrofatty tissue [39]. Besides, CD31^+^GLUT1^+^ blood vessels could be formed by HemSCs in immunodeficient mice [7]. Furthermore, CD133-sorting HemSCs from proliferating infantile hemangioma establish an mice model of hemangioma in vivo [40]. All of these findings suggest HemSCs serves as the cellular precursors of IHs. We thereby focused on HemSCs for the study of IHs with propranolol resistance.

CircRNAs have been less investigated in the tumorigenesis and progression of IHs. Here, we first explored the expression patterns and functional mechanisms of Circ_0000915 in IHs. It has been reported that STAT3 was highly activated in proliferating infantile hemangioma [41], and IL-6/STAT3 pathway play a pivotal role in tumorigenesis and progression of multiple cancer types [26, 42, 43]. STAT3 was thereby predicted and validated as the upstream regulator of Circ_0000915 in IHs. Given that circRNAs have been increasingly reported to function by serving as miRNA sponges [44–46], miR-890 was identified as the downstream target miRNA of Circ_0000915. In line with the tumor-suppressive role acted by miR-890 in the present study, miR-890 was reported to inhibit proliferation and invasion and induced apoptosis in triple-negative breast cancer cells by targeting CD147 [47]; it was also demonstrated that LINC00662 promoted cell proliferation, migration and invasion of melanoma by sponging miR-890 to up-regulate ELK3 [48]. Moreover, we further confirmed that RNF187 was a targetted by miR-890. RNF187, a E3 ubiquitin ligase to enable ubiquitin-protein transferase activity, has been widely reported to be an oncogene in multiple cancer types. For example, Chen et al. demonstrated overexpression of RNF187 induced cell EMT and apoptosis resistance in NSCLC [49]; Shi et al. revealed high level of RNF187 contributed to progression and drug resistance of osteosarcoma [50]; Liu et al. identified an essential oncogenic role of RNF187 in metastasis of hepatocellular carcinoma [51]. All these previous literatures strongly implied Circ_0000915 may play a pivotal role in IHs, which has driven us to make a further study in this work.

## Conclusion

**Fig. 9:**
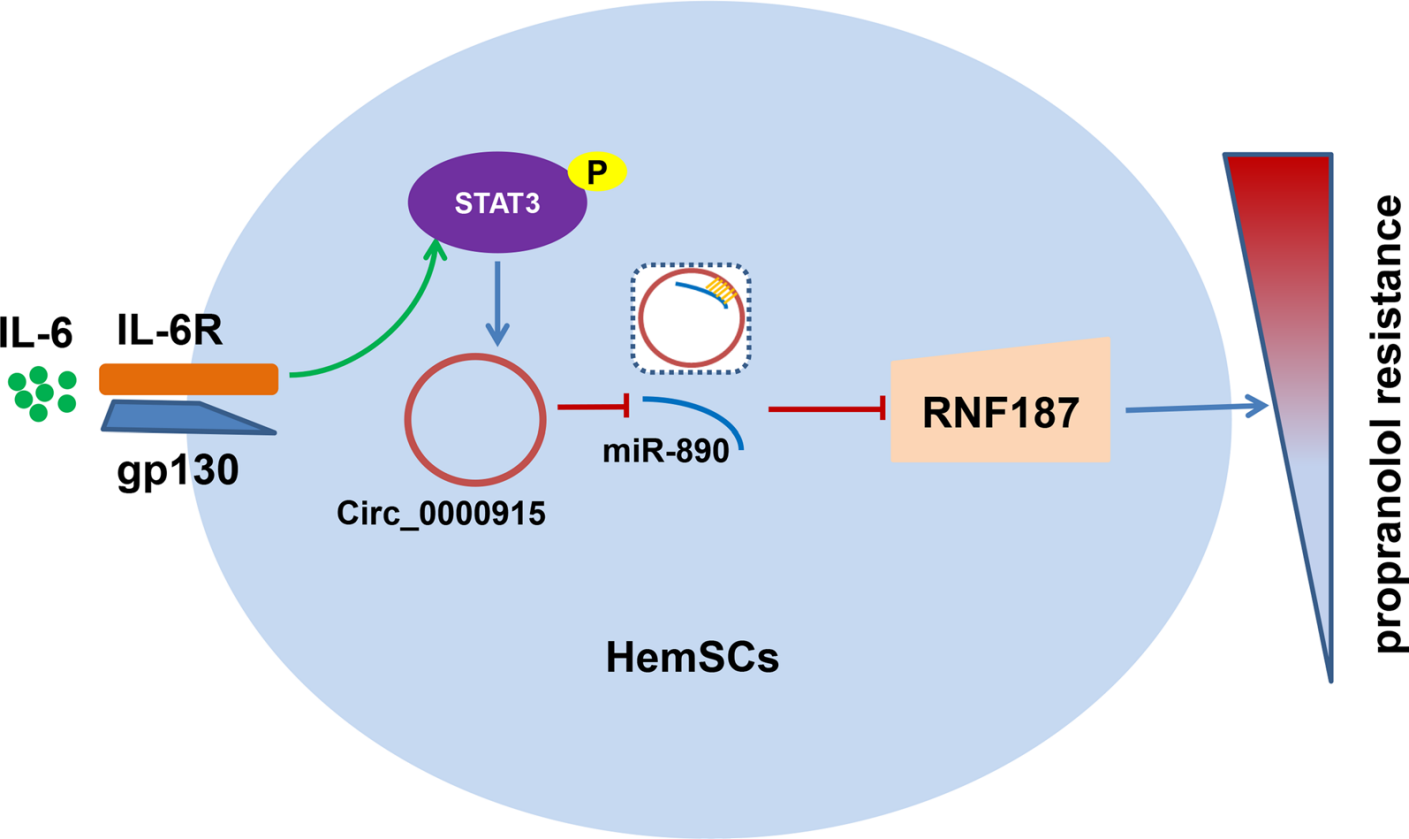
Schematic working model

In conclusion, we revealed a novel IL-6/STAT3/Circ_0000915/miR-890/RNF187 signal axis, which facilitated propranolol resistance of IHs in this study. Expression of Circ_0000915 was transcriptionally enhanced by IL-6-activated STAT3. We also demonstrated Circ_0000915 accelerated propranolol resistance of HemSCs via competing the same biding site at miR-890 with RNF187, resulting in release of inhibitory effects of miR-890 on RNF187, which consequently led to upregulation of RNF187 (Fig. 9). Besides, Circ_0000915 and RNF187 were up-regulated in IH tissues, especially in IH tissues from propranolol-resistant patients. MiR-890 exhibited an inverse expression pattern. Circ_0000915, miR-890 and RNF187 may thereby serve as prognostic indictors and potential therapeutic targets for IH patients with propranolol resistance.

## Supplementary Information

**Additional file 1**. Oligomers used in the study.

**Additional file 2**. Patient’s characteristics and response to propranolol.

## Data Availability

The original contributions presented in the study are included in the article/supplementary material, further inquiries can be directed to the corresponding author/s.
