# Supplementary material for: Circular RNA hsa_circ_0000915 promotes propranolol resistance of hemangioma stem cells in infantile haemangiomas
Source: Hum Genomics. 2022 Sep 27;16:43. doi: 10.1186/s40246-022-00416-w (PMC9513930; doi:10.1186/s40246-022-00416-w)
Supplement: Supplementary file 1 — Additional file 1. Oligomers used in the study. [file 40246_2022_416_MOESM1_ESM.docx]

**Table 1. Oligomers used in this study**

| Name | Application | Sequence |
| --- | --- | --- |
| Q-Circ_0000915-F | qRT-PCR | AACGGGCTGTTGAGGAAGAAG |
| Q-Circ_0000915-R | qRT-PCR | CGTCCATGAGTGGGACACTGA |
| Q-Circ_0000915-promoter-F | qRT-PCR | AACTGTGTGGGGGATGGGA |
| Q-Circ_0000915-promoter-R | qRT-PCR | GCTCTGGGGTTGCTGAATG |
| Q-RNF187-F | qRT-PCR | TGGAAATCATGAGAACTTG |
| Q-RNF187-R | qRT-PCR | ACGGTCCATCACGTGTCC |
| Q-Cyclin D1-F | qRT-PCR | CAAATGGAGCTGCTCCTGGTG |
| Q-Cyclin D1-R | qRT-PCR | CTTCGATCTGCTCCTGGCAGG |
| Q-PCNA-F | qRT-PCR | GGCGTGAACCTCACCAGTAT |
| Q-PCNA-R | qRT-PCR | TTCTCCTGGTTTGGTGCTTC |
| Q-β-ACTIN-F | qRT-PCR | CCTGTACGCCAACACAGTGC |
| Q-β-ACTIN-R | qRT-PCR | ATACTCCTGCTTGCTGATCC |
| U6-RT | RT | GTCGTATCCAGTGCAGGGTCCGAGGTATTCGCACTGGATACGACAAAATATGGAAC |
| miR-890-RT | RT | GTCGTATCCAGTGCAGGGTCCGAGGTATTCGCACTGGATACGACCAACTG |
| Q-miR-890-F | qRT-PCR | GCCGGCGCCCGAGCTCTGGCTC |
| Q-miR-890-R | qRT-PCR | TACTTGGAAAGGCATCAGTTG |
| Q-U6-F | qRT-PCR | GTGCTCGCTTCGGCAGCACAT |
| Q-U6-R | qRT-PCR | TACCTTGCGAAGTGCTTAAAC |
| si-Circ#1 | Cell transfection | GCCAGGTTCGAGCCGCCCGGT |
| si-Circ#2 | Cell transfection | ACTCATGGACGTGGGGGAGAC |
| si-STAT3#1 | Cell transfection | GAGAGATTGACCAGCAGTATA |
| si-STAT3#2 | Cell transfection | GCAAAGAAGGAGGCGTCACTT |
| si-RNF187#1 | Cell transfection | GGAAATCATGAGAAAGGACTT |
| si-RNF187#2 | Cell transfection | GAAGGCACTGACCGACTACAA |
| miR-890-mimics | Cell transfection | UACUUGGAAAGGCAUCAGUUGtt |
| miR-890-inhibitor | Cell transfection | CAACUGAUGCCUUUCCAAGUAtt |
| Circ_0000915-clone-F | plasmid construction | AATT GAATTC TGAAATATGCTATCTTAC AG GGAACGGGCTGTTGAGGA |
| Circ_0000915-clone-R | plasmid construction | AATT GGATCC TCAAGAAAAAATATATTC AC CTGCCTTGGGGGCCGTAGC |
| Circ_0000915-wt-F | plasmid construction | AATT CTCGAG GGAACGGGCTGTTGAGGA |
| Circ_0000915-promoter-F | plasmid construction | AATT CTCGAG GAGCCGAGATTGCGCCACTGCAC |
| Circ_0000915-promoter-R | plasmid construction | AATT AAGCTT CGCTTGGCCCCGCCCCTGCCGTGCC |
| Circ_0000915-wt-R | plasmid construction | AATT GCGGCCGC CTGCCTTGGGGGCCGTAGC |
| Circ_0000915-mt-F | plasmid construction | ATGGTCACTGCTGACACGCACTA |
| Circ_0000915-mt-R | plasmid construction | TAGTGCGTGTCAGCAGTGACCAT |
| RNF187 3’UTR-wt-F | plasmid construction | AATT CTCGAG TGGCGCCAACCCGTGGCAG |
| RNF187 3’UTR-wt-R | plasmid construction | AATT GCGGCCGC TGTGGAATCAAAATGTTGATTT |
| RNF187 3’UTR-mt-F | plasmid construction | CCCGGCCCAGGCAAATCGCACTC |
| RNF187 3’UTR-mt-R | plasmid construction | GAGTGCGATTTGCCTGGGCCGGG |
| Oligo probe-1 | RNA pull down | （biotin-）GTACATCTGCAGACGTCGCTGG |
| Circ_0000915 probe-1 | RNA pull down | （biotin-）CCAGCGACGTCTGCAGATGTAC |
| Oligo probe-2 | RNA pull down | （biotin-）GACGTCATCCAGGCCCTGGATC |
| Circ_0000915 probe-2 | RNA pull down | （biotin-）GATCCAGGGCCTGGATGACGTC |
| Oligo probe-3 | RNA pull down | （biotin-）CGGCCATGGTCACTGCTGACTCC |
| Circ_0000915 probe-3 | RNA pull down | （biotin-）GGAGTCAGCAGTGACCATGGCCG |
