# Supplementary material for: Circular RNA hsa_circ_0000915 promotes propranolol resistance of hemangioma stem cells in infantile haemangiomas
Source: Hum Genomics. 2022 Sep 27;16:43. doi: 10.1186/s40246-022-00416-w (PMC9513930; doi:10.1186/s40246-022-00416-w)
Supplement: Supplementary file 2 — Additional file 2. Patient’s characteristics and response to propranolol. [file 40246_2022_416_MOESM2_ESM.docx]

**Supplementary Table 2. Patient’s characteristics and response to propranolol**

| **Case** | **Age(month)** | **Sex** | **Type** | **Location** | **Response to propranolol** |
| --- | --- | --- | --- | --- | --- |
| 1 | 3 | female | mixed | cheek | yes |
| 2 | 4 | female | mixed | forehead | yes |
| 3 | 6 | male | superfacial | buttock | yes |
| 4 | 12 | female | deep | cheek | no |
| 5 | 4 | female | deep | neck | yes |
| 6 | 4 | female | superfacial | forehead | no |
| 7 | 5 | male | superfacial | abdomen | yes |
| 8 | 7 | female | superfacial | nasal tip | no |
| 9 | 2 | female | mixed | buttock | yes |
| 10 | 5 | female | mixed | nasal tip | no |
| 11 | 7 | female | mixed | cheek | yes |
| 12 | 9 | female | mixed | periocular | yes |
| 13 | 16 | female | superfacial | abdomen | yes |
| 14 | 2 | female | superfacial | forehead | no |
| 15 | 3 | male | superfacial | periocular | no |
| 16 | 3 | female | superfacial | nasal tip | yes |
| 17 | 6 | female | superfacial | inferior lip | no |
| 18 | 7 | female | superfacial | cheek | yes |
| 19 | 3 | female | deep | neck | yes |
| 20 | 6 | female | deep | knee | no |
| 21 | 4 | female | deep | forehead | yes |
| 22 | 3 | female | mixed | knee | yes |
| 23 | 17 | female | mixed | abdomen | no |
| 24 | 5 | female | mixed | cheek | yes |
| 25 | 2 | female | mixed | nasal tip | no |
| 26 | 11 | female | mixed | buttock | yes |
| 27 | 9 | female | mixed | cheek | no |
| 28 | 16 | male | mixed | forehead | yes |
| 29 | 12 | male | mixed | cheek | no |
| 30 | 4 | male | mixed | neck | yes |
| 31 | 3 | female | mixed | nasal tip | no |
| 32 | 7 | female | superfacial | periocular | yes |
| 33 | 7 | female | superfacial | cheek | no |
| 34 | 8 | female | superfacial | abdomen | no |
| 35 | 16 | female | superfacial | cheek | no |
| 36 | 4 | female | superfacial | inferior lip | no |
| 37 | 6 | female | deep | forehead | yes |
| 38 | 8 | male | deep | forehead | yes |
| 39 | 14 | male | mixed | periocular | no |
| 40 | 8 | female | mixed | cheek | yes |
| 41 | 4 | female | mixed | cheek | yes |
| 42 | 2 | female | mixed | cheek | yes |
| 43 | 3 | female | mixed | forehead | no |
| 44 | 6 | female | mixed | abdomen | yes |
| 45 | 3 | male | superfacial | forehead | no |
| 46 | 5 | female | superfacial | nasal tip | no |
| 47 | 8 | female | superfacial | inferior lip | yes |
| 48 | 3 | female | deep | forehead | yes |
| 49 | 2 | male | mixed | nasal tip | yes |
| 50 | 9 | female | mixed | forehead | yes |
